# Acute hyperventilation increases the central venous-to-arterial PCO_2_ difference in stable septic shock patients

**DOI:** 10.1186/s13613-017-0258-5

**Published:** 2017-03-20

**Authors:** Jihad Mallat, Usman Mohammad, Malcolm Lemyze, Mehdi Meddour, Marie Jonard, Florent Pepy, Gaelle Gasan, Stephanie Barrailler, Johanna Temime, Nicolas Vangrunderbeeck, Laurent Tronchon, Didier Thevenin

**Affiliations:** 1 Department of Anesthesiology and Critical Care Medicine, Service de Réanimation polyvalente, Centre Hospitalier du Dr. Schaffner, 99 route de La Bassée, 62307 Lens Cedex, France; 2 Intensive Care Unit, Centre Hospitalier d’Arras, Boulevard Georges Besnier, 62022 Arras Cedex, France

**Keywords:** Acute hyperventilation, Oxygen consumption, Central venous-to-arterial CO_2_ tension gap, Central venous oxygen saturation, Septic shock

## Abstract

**Background:**

To evaluate the effects of acute hyperventilation on the central venous-to-arterial carbon dioxide tension difference (∆PCO_2_) in hemodynamically stable septic shock patients.

**Methods:**

Eighteen mechanically ventilated septic shock patients were prospectively included in the study. We measured cardiac index (CI), ∆PCO_2_, oxygen consumption (VO_2_), central venous oxygen saturation (ScvO_2_), and blood gas parameters, before and 30 min after an increase in alveolar ventilation (increased respiratory rate by 10 breaths/min).

**Results:**

Arterial pH increased significantly (from 7.35 ± 0.07 to 7.42 ± 0.09, *p* < 0.001) and arterial carbon dioxide tension decreased significantly (from 44.5 [41–48] to 34 [30–38] mmHg, *p* < 0.001) when respiratory rate was increased. A statistically significant increase in VO_2_ (from 93 [76–105] to 112 [95–134] mL/min/m^2^, *p* = 0.002) was observed in parallel with the increase in alveolar ventilation. While CI remained unchanged, acute hyperventilation led to a significant increase in ∆PCO_2_ (from 4.7 ± 1.0 to 7.0 ± 2.6 mmHg, *p* < 0.001) and a significant decrease in ScvO_2_ (from 73 ± 6 to 67 ± 8%, *p* < 0.001). A good correlation was found between changes in arterial pH and changes in VO_2_ (*r* = 0.67, *p* = 0.002). Interestingly, we found a strong association between the increase in VO_2_ and the increase in ∆PCO_2_ (*r* = 0.70, *p* = 0.001).

**Conclusions:**

Acute hyperventilation provoked a significant increase in ∆PCO_2_, which was the result of a significant increase in VO_2_ induced by hyperventilation. The clinician should be aware of the effects of acute elevation of alveolar ventilation on ∆PCO_2_.

**Electronic supplementary material:**

The online version of this article (doi:10.1186/s13613-017-0258-5) contains supplementary material, which is available to authorized users.

## Background

Awareness that tissue hypoperfusion is a key factor in the pathogenesis of the multiple organ failures has focused the attention on surrogate indicators of tissue perfusion in the critically ill patient [1, 2]. In this context, venous-to-arterial carbon dioxide tension difference (∆PCO_2_) has been proposed as a marker of tissue hypoperfusion in patients with septic shock [3–9]. In fact, the increase in ∆PCO_2_ that has been observed in low-flow states is mainly related to venous hypercapnia, rather than a decrease in arterial CO_2_ partial pressure (PaCO_2_), which can be explained by the tissue CO_2_ stagnation phenomenon. Indeed, due to the decrease in transit time, there is a higher than usual addition of CO_2_ per unit of blood passing the efferent microvessels, which leads to a rise in CO_2_ partial pressure in the venous blood (PvCO_2_) [10–12].

The reason for the preferred use of ∆PCO_2_ over PvCO_2_ as a marker of global tissue perfusion is that ∆PCO_2_ was found to be less influenced by changes in PaCO_2_ than PvCO_2_ [13–15]. However, acute changes in PaCO_2_ or arterial pH might have direct effects on microvascular tone and/or might induce variations in systemic oxygen consumption [15–18], and could then affect ∆PCO_2_. Recently, in healthy volunteers, acute hyperventilation was shown to be associated with an increase in the peripheral venous-to-arterial CO_2_ difference due to a reduction in peripheral blood flow induced by acute hypocapnia [19, 20]. Furthermore, Morel et al. [21] found, in a small study that included mechanically ventilated postoperative patients, that acute decreases in PaCO_2_ resulted in significant increases in ∆PCO_2_ without any change in cardiac output. Therefore, it is not clear so far what the impact of the rapid decrease in PaCO_2_ on ∆PCO_2_ is. This question is important because if PaCO_2_ or arterial pH fluctuations could influence ∆PCO_2_, this effect will have to be taken into account by the physician when interpreting ∆PCO_2_ at the bedside. The aim of this study was to investigate the impact of acute hyperventilation on ∆PCO_2_ in mechanically ventilated and hemodynamically stable septic shock patients.

## Methods

This prospective and observational study was conducted in a single general adult intensive care unit (ICU). The study was approved by our local institutional ethics committee (Comité d’éthique du Centre Hospitalier du Dr. Shaffner de Lens, France). Informed consent was obtained from each subject’s next of kin.

### Patients

The study included mechanically ventilated and hemodynamically stable septic shock patients. The diagnosis of septic shock was defined according to the criteria of the American College of Chest Physicians (ACCP)/Society of Critical Care Medicine (SCCM) Consensus Conference [22]. All patients had to be monitored by a transpulmonary thermodilution device (PiCCO, Pulsion Medical System, Munich, Germany) as part of routine management of septic shock in our ICU.

To avoid spontaneous breathing activity, patients remained sedated throughout the study via continuous infusions of propofol and remifentanil. Patients were ventilated in the control volume mode.

Exclusion criteria were pregnancy, age less than 18 years old, unstable hemodynamic condition (change in vasoactive drug dosage or fluid administration within 1 h preceding the protocol), high blood lactate levels (>2 mmol/L), and uncontrolled tachyarrhythmias (heart rate 140 beats/min).

### Measurements

Demographic data, septic shock etiology, the Simplified Acute Physiology Score (SAPS) II, and the Sequential Organ Failure Assessment (SOFA) scores were obtained on the day of enrollment.

Cardiac index (CI) was obtained with the PiCCO monitor by triplicate central venous injections, in either the internal jugular or subclavian vein, of 20 mL of iced 0.9% saline solution and recorded as the average of the three measurements. In cases where the discrepancy in CI measurements was >10%, the measurement was repeated two more times (five times in total) with elimination of the highest and the lowest results.

Arterial and central venous blood gases analysis and arterial lactate levels were measured using the GEM Premier 4000 (Instrumentation Laboratory Co, Paris, France). To ensure accurate measurement, the blood gas analyzer was calibrated several times a day. Central venous blood was obtained from a central venous catheter with the tip confirmed to be in the superior vena cava at the entrance, or in the right atrium, by X-ray. ∆PCO_2_ was calculated as the difference between central venous carbon dioxide tension (PcvCO_2_) and arterial carbon dioxide tension (PaCO_2_). Arterial oxygen content was calculated as CaO_2_ (mL) = 1.34 × Hb (g/dL) × SaO_2_ + 0.003 × PaO_2_ (mmHg), where SaO_2_ is the oxygen saturation of arterial blood, Hb the hemoglobin concentration, and PaO_2_ the arterial oxygen tension. Central venous oxygen content was calculated as CcvO_2_ (mL) = 1.34 × Hb (g/dL) × ScvO_2_ + 0.003 × PcvO_2_ (mmHg), where PcvO_2_ is the central venous oxygen tension and ScvO_2_ the central venous oxygen saturation. DO_2_ was calculated by using the formula: DO_2_ (mL/min/m^2^) = CaO_2_ × CI × 10. VO_2_ was calculated using the following formula: VO_2_ (mL/m^2^) = CI × (CaO_2_ − CcvO_2_) × 10. Oxygen extraction was defined as: OE = VO_2_/DO_2_.

Heart rate (HR), mean arterial pressure (MAP), minute ventilation, respiratory rate, body temperature, and fractional inspired oxygen level were also recorded.

### Study protocol

Patients were in steady state defined as less than 10% variation in HR, MAP, CI, and SaO_2_ over a 60-min period before baseline measurements were initiated. Each patient was quiet and well adapted to the respirator. Fluid, doses of the vasopressor, and sedation drugs were kept constant in the hour preceding the measurements and throughout the study period. Variations in body temperature must have been <±0.5 °C. Enteral and/or parenteral nutrition were continued and remained unchanged during the data collection period.

At baseline, a first set of measurements was performed, including hemodynamic and tissue oxygenation variables (HR, MAP, CI, VO_2_, ScvO_2_), arterial lactate level, ∆PCO_2_, respiratory rate, and minute ventilation. Alveolar ventilation was then increased by raising the respiratory rate by 10 breaths/min (hyperventilation period). The inspiratory time was decreased to avoid the generation of an intrinsic positive end-expiratory pressure (PEEP) and to keep the level of plateau pressure constant throughout the study period. Also, the external PEEP remained unchanged. After 30 min of stabilization, a second set of measurement was recorded, including the same hemodynamic, respiratory, and tissue oxygenation variables (Additional file 1).

Changes in variables induced by the increase in alveolar ventilation were expressed as relative changes: [(variable after − variable before)/variable before] × 100.

### Statistical analysis

Data are presented as mean ± SD or as median (25–75%, interquartile range). Normality was evaluated using the Shapiro–Wilk test. Comparisons of variables between before versus after increase in alveolar ventilation were assessed using Student’s paired *t* test or Wilcoxon test, as appropriate. Linear correlations were tested using the Pearson or the Spearman test, as appropriate. The McNemar’s test was used to compare two paired proportions.

In a previous study [23], we found that the smallest detectable difference for ∆PCO_2_ was 2.0 mmHg. The smallest detectable difference is the minimum change (in absolute value) that needs to be measured by a laboratory analyzer in order to recognize a real change in measurement. Thus, for a power of 90% and α risk of 0.05, a sample size of 17 was required to detect a mean difference of 2.0 mmHg in ∆PCO_2_ with a standard deviation of 2.35 mmHg [23].

Statistical analysis was performed using STATA 14.0 (StataCorp LP, College Station, Texas, USA). *p* < 0.05 was considered statistically significant. All reported *p* values are 2-sided.

## Results

Eighteen septic shock patients were prospectively included in this study. Basic characteristics of the cohort are presented in Table 1. The principal source of infection was pneumonia (61%) with ICU mortality rate of 39%. All patients were sedated, mechanically ventilated without spontaneous breathing, and hemodynamically stable at their inclusions. No changes in vasopressor therapy and sedation level occurred during the observation period.

**Table 1:** Baseline characteristics of the patients (*n* = 18)

Age (mean ± SD, years)	60 ± 10
Gender (men/women)	8/10
Body mass index [median (IQR), kg/m^2^]	26.5 [25.6–29.7]
SAPS II	54 ± 21
Admission SOFA score (mean ± SD)	11 ± 3
ICU mortality [*n* (%)]	7 (39)
FiO_2_ (mean ± SD, %)	50 ± 20
Hemoglobin [median (IQR), g/dL]	9.7 [9.0–10.2]
Norepinephrine [*n* (%)]	18 (100)
Norepinephrine [median (IQR), µg/kg/min)	0.26 [0.15–0.40]
Infection source [*n* (%)]	
Pneumonia	11 (61)
Peritonitis	4 (22)
Urinary tract infection	2 (11)
Catheter/bloodstream	1 (6)
Mechanical ventilation [*n* (%)]	18 (100)

*SAPS*, simplified acute physiologic score; SOFA, sequential organ failure assessment; ICU, intensive care unit; FiO_2_, fractional inspired oxygen level; IQR, interquartile range; SD, standard deviation

### Effect of acute hyperventilation on blood gases, metabolic, and hemodynamic variables

Acute hyperventilation induced a significant increase in arterial pH and a significant decrease in PaCO_2_ (Table 2). Changes in arterial pH and PaCO_2_ were paralleled by a statistically significant increase in VO_2_ (Table 2). Cardiac index, heart rate, and DO_2_ remained unaffected.

**Table 2:** Blood gases, hemodynamic, ventilation, and metabolic parameters before and after hyperventilation

	Baseline	Hyperventilation	*p* value
PaCO_2_ [median (IQR), mmHg]	44 [41–48]	34 [30–38]	<0.001
PcvCO_2_ [median (IQR), mmHg]	49 [46–53]	42 [38–44]	<0.001
∆PCO_2_ (mean ± SD, mmHg)	4.7 ± 1.0	7.0 ± 2.6	<0.001
∆PCO_2_ ≤ 6 mmHg [*n* (%)]	17 (94.4)	8 (44.4)	0.004
Arterial pH (mean ± SD)	7.35 ± 0.07	7.42 ± 0.09	<0.001
Central venous pH (mean ± SD)	7.33 ± 0.07	7.39 ± 0.08	<0.001
Bicarbonate (mean ± SD, mmol/L)	25.6 ± 1.8	24.9 ± 1.6	<0.001
Base excess (mean ± SD, mmol/L)	0.1 ± 2.1	0.6 ± 2.1	0.002
PaO_2_/FiO_2_ (mean ± SD, mmHg)	210 ± 88	205 ± 92	0.64
CI (mean ± SD, L/min/m^2^)	3.03 ± 0.79	3.00 ± 0.78	0.27
Heart rate [median (IQR), beats/min]	85 [79–90]	86 [78–88]	0.70
Stroke index (mean ± SD, mL/m^2^)	36.2 ± 10.1	35.4 ± 10.3	0.25
MAP (mean ± SD, mmHg)	77 ± 8	79 ± 10	0.10
ScvO_2_ (mean ± SD, %)	73 ± 6	67 ± 8	<0.001
DO_2_ (mean ± SD, mL/min/m^2^)	396 ± 121	395 ± 114	0.62
VO_2_ [median (IQR), mL/min/m^2^]	93 [76–105]	112 [95–134]	0.002
OE (mean ± SD, %)	24.5 ± 5.8	31.4 ± 8.1	<0.001
Lactate (mean ± SD, mmol/L)	1.15 ± 0.47	1.35 ± 0.50	<0.001
SVRI (mean ± SD, dynes s/m^2^/cm^5^)	1645 ± 379	1940 ± 464	<0.001
Tidal volume [median (IQR), mL]	455 [430–500]	450 [430–500]	0.08
Respiratory rate [median (IQR), breaths/min]	20 [18–20]	30 [28–30]	<0.001
Minute ventilation (mean ± SD, L/min)	8.3 ± 1.6	12.6 ± 2.2	<0.001
Plateau pressure (mean ± SD, cm H_2_O)	23.5 ± 3.7	23.1 ± 4.7	0.60
Temperature (mean ± SD, °C)	37.1 ± 0.6	37.2 ± 0.6	0.21

PaCO_2_, partial arterial carbon dioxide tension; ∆PCO_2_, venous–arterial carbon dioxide tension difference; PaO_2_, partial arterial oxygen tension; FiO_2_, fractional inspired oxygen level; CI, cardiac index; MAP, mean arterial pressure; ScvO_2_, central venous oxygen saturation; DO_2_, oxygen delivery; VO_2_, oxygen consumption; OE, oxygen extraction; SVRI, systemic vascular resistance index; IQR, interquartile range; SD, standard deviation

Acute hyperventilation led to a significant increase in ∆PCO_2_, which resulted in a significant decrease in the number of patients with normal ∆PCO_2_ value (∆PCO_2_ ≤ 6 mmHg) (Table 2). Furthermore, we observed a significant reduction in ScvO_2_ in parallel to the increase in alveolar ventilation. Interestingly, lactate levels significantly increased when minute ventilation was increased (Table 2). Abrupt elevation of alveolar ventilation was also associated with a significant increase in systemic vascular resistance index (Table 2).

### Correlation analysis

**Fig. 1:**
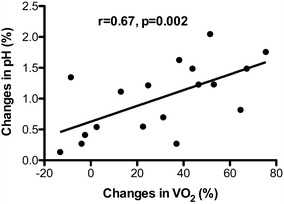
Relationship between changes in VO_2_ and changes in pH induced by the increase in alveolar ventilation

We found a good correlation between the increase in arterial pH induced by hyperventilation and the increase in VO_2_ (*r* = 0.67, *p* = 0.002) (Fig. 1). Changes in PaCO_2_ and VO_2_ were also moderately correlated (*r* = −0.54, *p* = 0.02).

**Fig. 2:**
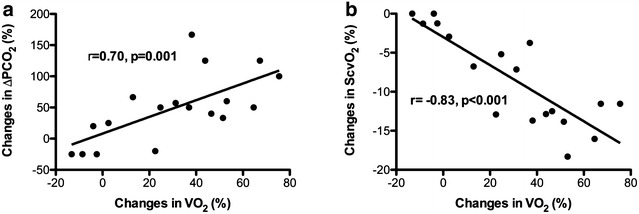
Relationship between changes in VO_2_ and the changes in ∆PCO_2_ (**a**) and ScvO_2_ (**b**) between after and before the increase in alveolar ventilation

The increase in ∆PCO_2_ observed after the elevation of alveolar ventilation was strongly associated with the increase in VO_2_ (*r* = 0.70, *p* = 0.001) (Fig. 2a). Also, a strong relationship was found between the increase in VO_2_ induced by hyperventilation and the decrease in ScvO_2_ (*r* = −0.83, *p* < 0.001) (Fig. 2b).

We found that the increase in lactate levels after the acute increase in alveolar ventilation significantly correlated with the increase in VO_2_ (*r* = 0.54, *p* = 0.02).

Hyperventilation-induced increase in systemic vascular resistance index did not correlate with the change in ∆PCO_2_ (*r* = −0.20, *p* = 0.41), or the change in ScvO_2_ (*r* = −0.13, *p* = 0.62).

## Discussion

The main findings of our study were as follows: (1) acute increase in alveolar ventilation resulted in a significant increase in ∆PCO_2_ accompanied with a significant decrease in ScvO_2_; (2) these changes were linked to a significant increase in oxygen consumption induced by acute hyperventilation.

Early identification and treatment of tissue hypoperfusion are critical factors in the management of septic shock patients. In this regard, ∆PCO_2_ has been considered as a marker that reflects the adequacy of tissue perfusion in septic shock states [3–9]. Increased ∆PCO_2_ is associated with venous hypercapnia, which is explained by the low-flow-induced CO_2_ stagnation phenomenon [11, 12]. Venous hypercapnia results from insufficient elimination of the CO_2_ produced by peripheral tissues, secondary to reductions in systemic and microcirculatory blood flow. However, under spontaneous breathing, hyperventilation may decrease PaCO_2_ and thus may preclude the CO_2_ stagnation-induced increase in PvCO_2_ [24]. Because alveolar hyperventilation would decrease both arterial and venous PCO_2_ without eliminating the increased venous-to-arterial PCO_2_ gap, it is recommended to assess ∆PCO_2_ rather than only monitor PvCO_2_ as a global marker of tissue perfusion [25].

However, a few studies have assessed the effects of acute hyperventilation on ∆PCO_2_ in critically ill patients [13, 14, 21]. We found that the acute increase in alveolar ventilation led to a significant increase in ∆PCO_2_ with an amplitude (2.2 mmHg) that was larger than its smallest detectable difference (2.0 mmHg) [23]. In addition, when the changes in ∆PCO_2_ are expressed as relative changes, acute hyperventilation induced a significant increase in ∆PCO_2_ with a magnitude (48.5%) that was also greater than its least significant change (32.4%) [23], which is the minimum change that needs to be measured by a laboratory analyzer in order to recognize a real change in measurement. In other words, the observed increase in ∆PCO_2_ can be considered as a true change and was not due to a random variation. Our findings are in agreement with the results of Morel et al. [21]. Indeed, these authors studied the effects of an acute decrease in PaCO_2_, obtained by increasing the respiratory rate, on ∆PCO_2_ in mechanically ventilated post-cardiac surgery patients. They found that acute hyperventilation provoked a significant increase in ∆PCO_2_ (from 4.2 ± 1.8 to 7.6 ± 1.7 mmHg), while the cardiac index was unaffected. In that study [21], ScvO_2_ also decreased in parallel with the increase in alveolar ventilation. Furthermore, in an animal study [16], the gradient between gastric mucosal PCO_2_ and PaCO_2_ (indicator of gut perfusion), obtained with gastric tonometry, increased significantly after hyperventilation. However, our results disagree with those of a previous study [13] that found no impact of hyperventilation on mixed venous-to-arterial PCO_2_ difference in mechanically ventilated patients. In that study, the increase in alveolar ventilation was obtained very progressively by increasing the tidal volume from 7 to 10 mL/kg over a period of 3 h, which might explain the absence of changes in mixed venous-to-arterial PCO_2_ difference. Also, the mean cardiac index at baseline was high (4.55 ± 0.90 mL/min/m^2^), which would have prevented any increase in mixed venous-to-arterial PCO_2_ difference by washing out any addition of CO_2_ from the peripheral circulation.

Several mechanisms can be suggested to explain the increase in ∆PCO_2_ observed in our study. A first potential explanation is that acute hyperventilation provoked the increase in systemic oxygen consumption and therefore CO_2_ production. Thus, for a given venous blood flow, the increase in tissue CO_2_ production should lessen the decrease in PcvCO_2_ (induced by hyperventilation) relatively to the decrease in PaCO_2_, leading to a rise in ∆PCO_2_. We believe that such a mechanism may have contributed to the increase in ∆PCO_2_ after acute hyperventilation in our study. Indeed, we observed a strong correlation between the increases in VO_2_ between before and after hyperventilation and the increases in ∆PCO_2_ (Fig. 2a). Also, the magnitude of the decrease in PcvCO_2_ after hyperventilation was significantly less than the decrease in PaCO_2_ (−16.5 ± 4.8 vs. −22.7 ± 5.5%, *p* < 0.001, respectively), explaining the observed increase in ∆PCO_2_. Similarly, the reduction in ScvO_2_ found after hyperventilation can be explained by the increase in VO_2_. It is unlikely that the increase in VO_2_ with hyperventilation was a result of an unstable state because of the lack of hemodynamic and temperature differences (Table 2), and the absence of changes in vasopressor and sedation drugs during the study period. We think that the observed increase in VO_2_ was induced by acute hyperventilation since we found a good association between changes in pH and changes in VO_2_ (Fig. 1). Acute respiratory alkalosis has been found, in some experiments in animals and humans, to increase VO_2_ and CO_2_ production independently of any significant hemodynamic changes [17, 18, 26, 27]. Indeed, hyperventilation alkalosis, in mechanically ventilated dogs, increased VO_2_ by 10–25% [17, 18]. In anesthetized paralyzed patients, contradictory findings were observed with some authors reporting a significant increase in whole-body VO_2_ [27], whereas others failed to demonstrate any significant variation [14]. Recently, Morel et al. [20], reported a twofold increase in VO_2_ in healthy volunteers with hypocapnic condition compared to hypercapnic condition for the same minute volume, suggesting a possible contribution of this mechanism to the observed increase in peripheral venous-to-arterial CO_2_ difference after induced acute respiratory alkalosis. The mechanism by which an acute respiratory alkalosis stimulates oxygen consumption is unclear and may involve many intracellular processes. A decrease in intracellular hydrogen ion concentration may stimulate the activity of phosphofructokinase, a key enzyme in the glycolytic cycle, which could result in increased intracellular adenosine triphosphate (ATP) hydrolysis and increased VO_2_ [28, 29]. Interestingly, we found a significant increase in lactate level induced by acute hyperventilation (Table 2). This finding could be an indirect marker supporting the activation of the phosphofructokinase enzyme and the increased rate of glycolysis in our study. Indeed, several studies reported increased lactate production with alkalosis [30, 31], reflecting increased glycolysis.

A second possibility is that acute hypocapnia resulted in systemic vasoconstriction, thus decreasing the elimination of the total CO_2_ produced by the peripheral tissues, and increasing the ∆PCO_2_. It has been shown that acute hypocapnia induces vasoconstrictive responses in various organs [14, 32, 33]. In healthy volunteers, Umeda et al. [19] observed that acute hyperventilation decreased both the minimal and mean flow velocity in the radial artery assessed by Doppler echography. The authors concluded that the decrease in mean blood flow, which was the result of increased vascular tone induced by hyperventilation, was responsible for the rise in peripheral venous-to-arterial CO_2_ difference that they observed after acute hyperventilation. Similarly, Morel et al. [20] found a significant drop in the skin microcirculatory blood flow of healthy volunteers, evaluated with in vivo reflectance confocal microscopy, secondary to acute hypocapnia. In our study, we observed a significant increase in systemic vascular resistance in parallel with the elevation of alveolar ventilation (Table 2). Nevertheless, changes in systemic vascular resistance were not significantly correlated with changes in ∆PCO_2_ nor with changes in ScvO_2_, which suggests, indirectly, a minimal participation of this mechanism to the increase in ∆PCO_2_. However, since we did not specifically evaluate the microcirculation we cannot eliminate or confirm the contribution of the vasoconstrictive mechanism to the observed increase in ∆PCO_2_ secondary to acute hyperventilation.

A third possibility of the increase in ∆PCO_2_ is that acute hyperventilation could induce variations in the PCO_2_/CO_2_ content relationship. This mechanism is, however, unlikely to have occurred in our patients. Indeed, the relationship between CO_2_ content and PCO_2_, which is curvilinear rather than linear, is influenced by many factors such as the degree of metabolic acidosis, the hematocrit, and the oxygen saturation (Haldane effect) [12, 34]. Our patients did not have metabolic acidosis, and acute hyperventilation did not change the base excess meaningfully (Table 2). Although venous oxygen saturation decreased significantly after acute hyperventilation, it is unlikely that this change could have affected the PCO_2_/CO_2_ content relationship, because first, it was not large; in this extent as stressed by Jakob et al. [35], changes in ∆PCO_2_ might not parallel changes in CO_2_ content differences under conditions of very low values of venous oxygen saturation (<30%), which was not the case in our patients. Second, if Haldane effect had affected the PCO_2_/CO_2_ content relationship, it would have resulted in a decrease in ∆PCO_2_, rather than an increase in ∆PCO_2_ [36].

Our results are of clinical importance. Indeed, changes in ventilator settings are regularly needed in mechanically ventilated patients. Since ∆PCO_2_ is now widely recognized as a valuable marker to evaluate tissue perfusion in septic shock, a clinician should be aware that acute changes in pH or PaCO_2_ induced by hyperventilation could impact ∆PCO_2_ independently of changes in tissue perfusion. These findings should not dismiss the clinical value of ∆PCO_2_ as a marker to detect tissue perfusion derangements. On the contrary, our results highlighted the usefulness of ∆PCO_2_, as an index of VCO_2_/cardiac output ratio, to detect the imbalance between the relative increase in VCO_2_ and the blood flow, whatever the mechanism of this imbalance is (increases in oxygen consumption [37, 38] or tissue hypoperfusion [9]).

We acknowledge some limitations to our study. First, the number of patients studied was small, but the study was sufficiently powered to detect a real change in ∆PCO_2_ induced by hyperventilation. Second, the study was performed in a sample of septic shock patients from a single center, potentially limiting the generalizability of the results. However, our results confirm those of a previous study performed in a different patient population (post-cardiovascular surgery patients) [21]. Third, VO_2_ was calculated from central venous oxygen saturation and not from mixed venous oxygen saturation or measured by indirect calorimetry, what might limit its accuracy. However, in our study, we were interested in investigating the changes in VO_2_ induced by acute hyperventilation rather than by its absolute value. Furthermore, it has recently been demonstrated that calculating the oxygen-derived variables from the central venous blood allowed the detection of global tissue hypoxia in critically ill patients [39, 40]. Finally, we did not evaluate the microcirculation, and thus, we were incapable of drawing any conclusions about the effects of acute hyperventilation on the local vascular tone and its relationship to ∆PCO_2_.

## Conclusion

In stable septic shock patients, acute hyperventilation leads to a significant increase in ∆PCO_2_, which is associated with an increase in VO_2_, also induced by hyperventilation. Microcirculatory dysfunction is a key pathological mechanism involved in septic shock patients and could, also, be exacerbated by acute changes in alveolar ventilation. This finding should be taken into account when interpreting ∆PCO_2_ at the bedside. Further studies are needed to investigate the effects of acute hyperventilation on systemic oxygen consumption and local vascular tone.

## Additional file

**Additional file 1.** Manuscript data set.

## Abbreviations

CI: cardiac index
DO_2_: oxygen delivery
VO_2_: oxygen consumption
ScvO_2_: central venous oxygen saturation
∆PCO_2_: central venous-to-arterial carbon dioxide tension difference
CaO_2_: oxygen content
CcvO_2_: central venous oxygen content
PaCO_2_: arterial carbon dioxide tension
PcvCO_2_: central venous carbon dioxide tension
SaO_2_: arterial oxygen saturation
HR: heart rate
MAP: mean arterial pressure
Hb: hemoglobin
ICU: intensive care unit
PEEP: end-expiratory pressure
OE: oxygen extraction
